# Association of iron status indicators with thyroid hormone concentrations during pregnancy: a systematic review and meta-analysis

**DOI:** 10.3389/fendo.2025.1533169

**Published:** 2025-01-28

**Authors:** Mohammadamin Parsaei, Mohadese Dashtkoohi, Elahe Amirkhalili, Mohammadreza Chashmyazdan, Tim I. M. Korevaar, Pantea Nazeri

**Affiliations:** ^1^ Breastfeeding Research Center, Family Health Research Institute, Tehran University of Medical Sciences, Tehran, Iran; ^2^ Vali-e-Asr Reproductive Health Research Center, Family Health Research Institute, Tehran University of Medical Sciences, Tehran, Iran; ^3^ Department of Epidemiology and Statistics, School of Public Health, Tehran University of Medical Sciences, Tehran, Iran; ^4^ Department of Basic Medical Sciences, Khoy University of Medical Sciences, Khoy, Iran; ^5^ Department of Internal Medicine, Erasmus University Medical Center, Rotterdam, Netherlands; ^6^ Academic Center for Thyroid Diseases, Erasmus University Medical Center, Rotterdam, Netherlands

**Keywords:** hemoglobin, iron, pregnancy, serum ferritin, thyroid hormones

## Abstract

**Background:**

Maternal thyroid hormones play a vital role in fetal development, and imbalances can lead to adverse outcomes. Iron deficiency may impair thyroid function due to iron’s essential role in iodine oxidation during thyroid hormone synthesis. This review examines the relationship between various indicators of maternal iron status and thyroid function during pregnancy.

**Methods:**

We conducted a systematic search in MEDLINE/PubMed, Web of Science, Embase, Scopus, and the Cochrane Library for studies published up to 2023. Meta-analyses determined pooled thyroid hormone levels in patients with and without iron deficiency, using serum ferritin (cut-off = 30 µg/L) and hemoglobin (cut-off = 11 g/dL). Meta-regression analyses examined linear relationships between iron status indicators and thyroid hormones.

**Results:**

Forty-seven studies involving 53,152 pregnant women were included. Meta-analysis showed no significant difference in thyroid-stimulating hormone, free T4, or total T4 when considering serum ferritin levels in iron-deficient versus iron-sufficient individuals. However, regarding hemoglobin levels, iron deficiency was associated with higher thyroid-stimulating hormone (2.31 mIU/L vs. 1.75 mIU/L) and lower free T4 (10.7 pmol/L vs. 13.3 pmol/L), but not total T4. Meta-regression revealed no significant associations between serum ferritin and thyroid hormones. Conversely, maternal hemoglobin levels were inversely associated with thyroid-stimulating hormone (P-value = 0.009) and directly associated with free T4 (P-value < 0.001), with no significant link to total T4.

**Conclusions:**

Maternal hemoglobin levels are more strongly correlated with thyroid function than serum ferritin levels. This suggests that monitoring hemoglobin could enhance the early detection and management of thyroid dysfunction during pregnancy.

**Systematic Review Registration:**

https://www.crd.york.ac.uk/PROSPERO, identifier CRD4202451820.

## 1 Introduction

Thyroid dysfunction is a common endocrine disorder during pregnancy, affecting approximately 2.5% of pregnancies (1). Various thyroid disorders can develop or worsen during pregnancy, potentially leading to adverse outcomes for both the mother and the fetus (2). These changes are primarily due to altered thyroid-stimulating hormone (TSH) and thyroid-binding protein levels, influenced by elevated maternal estrogen levels (3–5). For instance, maternal hypothyroidism is associated with increased risks of adverse fetal outcomes such as preeclampsia, low birth weight, and intellectual impairments. Conversely, hyperthyroidism can lead to fetal complications including tachycardia, growth restriction, prematurity, and stillbirths (6–8). Therefore, early detection and treatment of thyroid disorders in pregnancy are crucial.

Iron deficiency (ID), characterized by reduced extracellular iron in the bone marrow and serum ferritin (SF), is recognized as the most prevalent nutritional deficiency (9). Pregnant women are particularly susceptible to ID and its more severe form, ID anemia, due to the increased iron demands associated with expanded blood volume to support maternal physiological functions and fetal development (10). It is suggested that ID can adversely affect thyroid hormone synthesis due to its critical role in intracellular oxygen delivery within thyroid tissue, thereby disrupting related metabolic pathways (11). Iron is a component of thyroid peroxidase (TPO), which is essential for thyroid hormone biosynthesis (12). TPO catalyzes the oxidation of iodine, a process activated by TSH. Thus, ID may hinder TPO activity and thyroid metabolism, reducing thyroid hormone production (13).

While several studies have investigated the relationship between thyroid function and ID during pregnancy, this association remains inadequately established (14, 15). A systematic review of eight articles indicated that ID is associated with elevated levels of TSH and reduced levels of free thyroxine (FT4), as well as an increased prevalence of subclinical and overt hypothyroidism in pregnant women (9). Nonetheless, more research is needed to explore the relationship between maternal iron status indicators beyond SF and thyroid function during pregnancy (16, 17). For example, Hb is one of the most robust indicators of body iron status, and its association with maternal thyroid function has been studied extensively (6, 17). However, a comprehensive overview is still lacking.

This study aims to systematically review the association between various indicators of maternal iron status and thyroid function in pregnant women. We expect that the findings will provide valuable insights into the relationship between iron status and thyroid function during pregnancy. These insights could potentially inform guidelines for diagnosing and managing pregnancy-induced thyroid disorders by taking maternal iron status into account.

## 2 Materials and methods

### 2.1 Search strategy

This study followed the Preferred Reporting Items for Systematic Reviews and Meta-analyses (PRISMA) guidelines (18) and is registered in the International Prospective Register of Systematic Reviews (PROSPERO) under the registration code CRD42024518203. The research question for this systematic review was formulated using the Participants, Intervention, Comparators, Outcomes, Study design (PICOS) framework (19), as follows:

Population (P): Healthy pregnant women;

Intervention (I): Maternal iron status;

Comparators (C): ID vs. Iron sufficiency;

Outcomes (O): Maternal thyroid function parameters and thyroid disorders;

Study Design (S): Observational studies, i.e., prospective and retrospective cohorts, cross-sectional, and case-control studies.

We conducted an extensive literature search across multiple databases, including MEDLINE/PubMed, Web of Science, Embase, Scopus, and the Cochrane Library, covering publications up to 2023. Our search employed key terms such as ‘pregnancy,’ ‘pregnant women,’ ‘iron status,’ ‘iron deficiency,’ ‘iron markers,’ ‘iron levels,’ ‘thyroid function parameters,’ ‘thyroid hormones,’ ‘thyroid gland,’ and ‘thyroid disorders.’ Additionally, we performed a manual search by reviewing the reference lists of original articles and relevant reviews.

### 2.2 Study selection and data extraction

Following the searches, two independent investigators (MP and PN) meticulously screened the titles and abstracts of studies to exclude those that did not meet the eligibility criteria. Subsequently, they conducted a thorough review of the full texts of potentially relevant studies to determine their inclusion in the systematic review. The inclusion criteria encompassed the following aspects: 1) human studies, 2) healthy pregnant women as participants, 3) studies reporting iron status using at least one indicator, and 4) studies providing data on various thyroid parameters and frequency of thyroid disorders. Notably, we excluded studies involving pregnant women who were supplemented with iron/folic acid, as well as those with pregnancy complications.

In the case of included studies, we utilized a standardized form specifically designed for this review to extract relevant data. The extracted information included details such as the first author, year of publication, study country, the number of pregnant women investigated, pregnancy timing, iron status indicators, thyroid tests, and any potential correlations between maternal iron status and thyroid function parameters or thyroid disorders during pregnancy. To ensure accuracy, the extracted data underwent cross-checking, and any discrepancies were resolved through discussion or consultation with a third investigator (MD).

### 2.3 Quality assessment

We evaluated the quality of studies using the Newcastle-Ottawa Scale (20). Each study was assessed across the following domains: 1) sample population selection [scored on a scale of 0–4], 2) comparability of subjects in different outcome groups [scored on a scale of 0–3], and 3) appropriate outcome assessment [scored on a scale of 0–3]. Based on their Newcastle-Ottawa scores, studies were categorized as very good (9–10 points), good (7–8 points), satisfactory (5–6 points), or unsatisfactory (0–4 points).

### 2.4 Data synthesis and statistical analysis

In this study, thyroid function in pregnant women was evaluated using TSH, free T4 (FT4), and total T4 (TT4). The means and standard deviations (SDs) of these continuous variables were extracted from each included study for the meta-analysis. When mean and SD values were not explicitly reported, they were estimated from available medians, interquartile ranges (IQRs), ranges, or 95% confidence intervals (CIs) using established formulas (21, 22).

Data analysis was performed using Comprehensive Meta-Analysis (CMA) software version 2.2.064, with statistical significance set at a P-value < 0.05 and a 95% CI. Since most of the included studies assessed the association between SF or Hb levels and thyroid hormones, and only a few studies have investigated the role of other iron status indicators in relation to thyroid function, we focused on these two indicators in this meta-analysis. Meta-analyses were conducted to calculate the pooled thyroid hormone levels in pregnant women based on their Hb and SF levels. For studies examining SF, a cut-off of 30 µg/L was used to distinguish between iron sufficiency (above 30 µg/L) and ID (below 30 µg/L) (23). For studies reporting Hb levels, pregnant women were categorized into two groups with Hb levels above 11 mg/dl (indicating iron sufficiency) and below 11 mg/dl (indicating ID) (24). Heterogeneity was evaluated using Cochrane’s Q statistic and the *I*² index, with *I*² values exceeding 50% considered indicative of substantial heterogeneity. In the presence of significant heterogeneity, a random-effects model was applied to calculate pooled effects; otherwise, a fixed-effects model was planned. Forest plots were generated to visually represent the pooled mean and 95% CI of thyroid hormones in different subgroups of iron status. We evaluated the presence of publication bias by both Egger’s regression asymmetry test in addition to funnel plot (25). Additionally, in relevant cases, we used the trim and fill method to address publication bias. Corrected results were reported after trimming if they significantly differed from the results where publication bias was present (26).

Furthermore, a meta-regression model was applied to investigate the linear relationships between iron status indicators (SF and Hb) and the three thyroid hormones (TSH, FT4, and TT4) during pregnancy. For this purpose, CMA software version 2.2.064 was utilized, with statistical significance set at a p-value < 0.05. The analysis employed mixed-effects regression using the method of moments. This approach enabled the examination of the relationship between study-level covariates and effect sizes across the included studies. The meta-regression output included pooled β, standard errors (SEs), and P-values for the slope.

## 3 Results

### 3.1 Study selection

**Figure 1:**
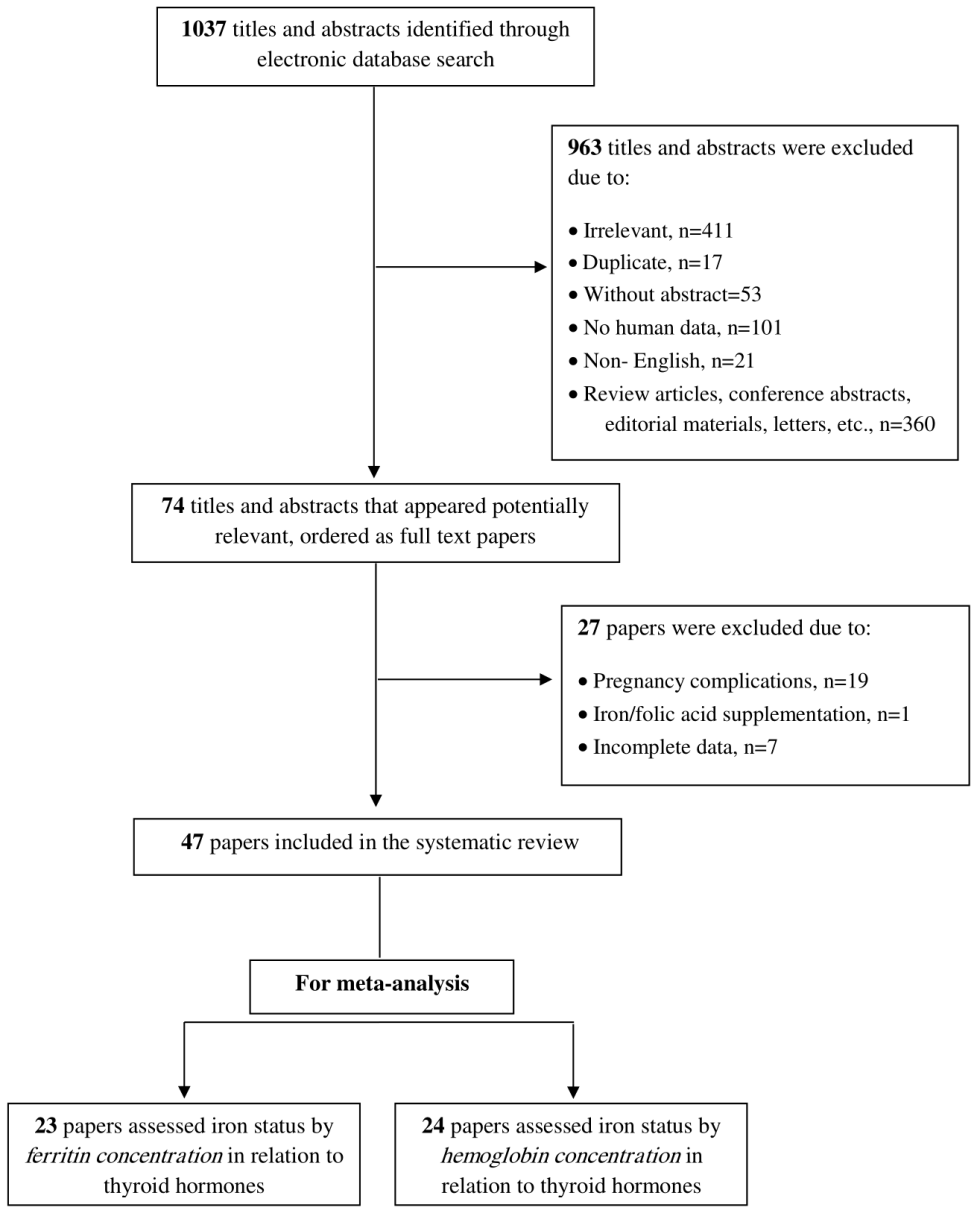
Flow diagram of the study selection process based on the PRISMA 2020 statement.

The systematic search yielded a total of 1,037 studies. After excluding 963 studies during the title and abstract screening process, 74 studies underwent full-text screening and were assessed for eligibility. Of these, 19 studies were excluded due to the inclusion of women with pregnancy-related complications, 7 were excluded due to incomplete data, and 1 was excluded due to iron and folic acid supplementation. Consequently, 47 studies successfully met the inclusion criteria and were systematically reviewed. **Figure 1** provides detailed information regarding the study selection process for this review.

### 3.2 Study characteristics

The geographical distribution of the reviewed studies is as follows: Eastern Asia (n=15), Southern Asia (n=14), Europe (n=9), the Middle East (n=6), Africa (n=1), North America (n=1), and South America (n=1). Most studies employed a cross-sectional design (n=31) (6, 12, 16, 27–54), while 13 studies utilized a prospective cohort design (17, 55–66) and three employed a retrospective cohort design (67–69). Sample sizes across the studies ranged from 28 to 14,043 pregnant women, culminating in a total of 53,152 patients included in this systematic review. Twenty studies focused on women in their first trimester of pregnancy (6, 12, 16, 29–36, 39, 45–47, 49, 53, 58, 60, 69), with four (38, 43, 50, 63) and seven studies (27, 37, 40, 52, 55, 61, 62) exclusively examining women in their second and third trimesters, respectively. Additionally, four studies included women in both the first and second trimesters (17, 41, 56, 68), two included women in the first and third trimesters (44, 48), and one study included women in both the second and third trimesters (66). Furthermore, nine studies encompassed women from all three trimesters of pregnancy (28, 42, 51, 54, 57, 59, 64, 65, 67).

The reported measures of iron status across the reviewed studies included: hemoglobin (Hb) (n=34), SF (n = 29), serum iron (SI) (n=8), mean corpuscular volume (MCV) (n=7), red blood cell (RBC) count (n=6), mean corpuscular hemoglobin (MCH) (n=5), transferrin receptor (TfR) (n=5), mean corpuscular hemoglobin concentration (MCHC) (n=4), hematocrit (Hct) (n=3), total iron-binding capacity (TIBC) (n=3), body iron store (BIS) (n=2), total body iron (TBI) (n=2), transferrin (Tf) (n=2), transferrin saturation (TS) (n=2), dietary intake recall (DIR) (n=1), erythropoietin (EP) (n=1), packed cell volume (PCV) (n=1), and urinary iron-binding capacity (UIBC) (n=1). The distribution of reported thyroid function measures included: TSH (n=45), FT4 (n=37), free T3 (FT3) (n=17), TT4 (n=16), anti-thyroid peroxidase antibody (TPO-Ab) (n=15), total T3 (TT3), anti-thyroglobulin antibody (Tg-Ab) (n=7), thyroglobulin (Tg) (n=3), thyroid volumes assessed via sonographic examination (n=2), and reverse T3 (r-T3) (n=1). More detailed information from each included study is provided in **Table 1**.

**Table 1:** Maternal iron status and thyroid parameters and thyroid disorders during pregnancy.

Study	Country	Design	Pregnant women, n	Trimester	Iron status indicators	Thyroid function tests	Thyroid disorders	Main findings
Geissler et al. (1979) (55)	Iran	PC	57	t3	Hb	FT4, TT4, TBG saturation	Hypothyroidism (35.1%)	No association between thyroid function and iron status was reported.
Price et al. (2001) (56)	UK	PC	70	t1, t2	SF	FT4, FT3, TSH	–	No association between thyroid function and iron status was reported.
Pathak et al. (2004) (27)	India	CS	283	t3	SF, DIR	TSH	–	TSH: ─ SF
Zimmermann et al. (2007) (28)	Switzerland	CS	365	t1, t2, t3	Hb, MCV, SF, TfR, BIS	TSH, TT4	–	TSH: ▴: TfR ▾: SF and BIS TT4: ▴: SF and BIS ▾: TfR
Larsson et al. (2008) (57)	Sweden	PC	52	t1, t2, t3	SF, SI, Tf	TSH	–	No association between thyroid function and iron status was reported.
Mahajan et al. (2008) (58)	USA	PC	300	t1	Hb, SF, EP	T3, r-T3, T4, TSH	–	T3: ↓ in severely anemic (Hb < 6.9 g/dl) than non-anemic mothers. T4, r-T3, and TSH: ↔
Jaiswal et al. (2014) (29)	India	CS	334	t1	Hb	FT3, TT3, FT4, TT4, TSH, Tg, TBG, TPO-Ab, Tg-Ab, Thyroid volumes	Subclinical hypothyroidism (9.2%) Hypothyroxinemia (5.2%) Overt hypothyroidism (3.7%) Subclinical hyperthyroidism (1.8%) Overt hyperthyroidism (0.3%)	TSH: ▾ Hb FT4: ▴ Hb * Hb levels were ↓ in women with hypothyroidism.
Joshi et al. (2014) (30)	India	CS	256	t1	Hb	FT4, TT4, TSH	–	No association between thyroid function and iron status was reported.
Refaat et al. (2014) (31)	Saudi Arabia	CS	500	t1	Hb, RBCs count, PCV, MCV, MCH, SI, SF, TIBC, TS	FT4, TSH	Subclinical hypothyroidism (20.4%) Overt hypothyroidism (6.4%) Hypothyroxinemia (4.8%) Hyperthyroidism (1.2%)	FT4: ▴ with RBCs count, Hb, and PCV TSH: ▴: TIBC ▾: SI, SF, and TS * RBCs count, Hb, SF, and TS were lower, and SI levels were ↑ in hypothyroidism and hypothyroxinemia than healthy controls.
Yu et al. (2015) (32)	China	CS	3340	t1	SF, TfR, TBI	FT4, TSH, TPO-Ab, TBI	Hypothyroxinemia (11.0%) Subclinical hypothyroidism (3.1%) Overt hyperthyroidism (1.0%) Subclinical hyperthyroidism (0.6%) Overt hypothyroidism (0.3%)	TSH: ▾ TBI FT4: ▴ TBI * In mothers with ID, FT4 level was ↓ and the prevalence of hypothyroxinemia was ↑.
DeZoysa et al. (2016) (59)	Sri Lanka	PC	425	t1, t2, t3	Hb, SF	FT4, TSH, Tg, Thyroid volumes	–	TSH: ▾ Hb FT4: ▾ SF
Li et al. (2016) (33)	China	CS	2581	t1	Hb, SF, RBCs count, Hct, MCH, MCHC	FT4, TT4, TSH, TPO-Ab	Subclinical hypothyroidism (12.1%) Overt hypothyroidism (9.3%) Overt hyperthyroidism (6.0%) Subclinical hyperthyroidism (4.2%)	FT4: ↓ in ID TT4: ↔ TSH: ↑ in ID TPO-Ab: ↑ in ID anemia * In mothers with ID, overt and subclinical hypothyroidism and TPO-Ab positivity was ↑.
Veltri et al. (2016) (34)	Belgium	CS	1900	t1	SF	FT4, TSH, TPO-Ab	Subclinical hypothyroidism (17%) Thyroid autoimmunity (8%)	FT4: ▴ SF TSH: ▾ SF * In mothers with ID, TSH was ↑, FT4 was ↓, and TPO-Ab was ↔. * In mothers with ID, subclinical hypothyroidism and thyroid autoimmunity (TPO-Ab > 60 kIU/L) were ↑.
Baghel et al. (2017) (35)	India	CS	40	t1	SI	FT3, FT4, TSH	–	No association between thyroid function and iron status was reported.
Fu et al. (2017) (36)	China	CS	1764	t1	Hb, SF	FT3, FT4, TSH	–	FT3: ▴ SF FT4: ─ SF TSH: ▾ SF * FT4 levels were ↓ in mothers with SF < 100 μg/L. * TSH levels were ↑ in mothers with SF < 20 μg/L.
Jiskani et al. (2017) (37)	Pakistan	CS	245	t3	Hb, SF	FT3, FT4, TSH	Hypothyroidism (22.8%) Hyperthyroidism (7.7%)	* In mothers with ID, prevalence of hypothyroidism and hyperthyroidism was ↑.
He et al. (2018) (38)	China	CS	209	t2	Hb, SI, SF, UIBC, TIBC, TS	FT4, TSH, TPO-Ab, Tg-Ab	–	FT4: ▴ SF TSH: ▾ SF TPO-Ab: ─ SF Tg-Ab: ─ SF
Rosario et al. (2018) (60)	Brazil	PC	596	t1	SF	FT4, TT4, TSH, TPO-Ab	Hypothyroxinemia (4.3%)	* In mothers with ID, hypothyroxinemia was ↑.
Teng et al. (2018) (67)	China	RC	1707	t1, t2, t3	SF, TfR, TBI	FT4, TSH, TPO-Ab, Tg-Ab	Hypothyroxinemia (t1: 1.8%, t2: 2.22%, t3: 2.91%) Subclinical hypothyroidism (t1: 1.6%, t2: 2.22%, t3: 2.27%) Overt hypothyroidism (t1: 0%, t2: 0.74%, t3: 0%)	FT4: t1 (n = 723): ▴ with SF and TBI, ▾ with TfR. t2 (n = 675): ▴ with SF and TBI, ▾ with TfR. 3. Third trimester (n = 309): ─ * In mothers with ID during their either t1 or t2 of pregnancy, FT4 levels were ↓ and prevalence of hypothyroxinemia was ↑, while in t3 ↔. * In mothers with diagnosed ID during t1, the serum FT4 levels of t2 and t3 were ↓.
Zhang et al. (2019) (39)	China	CS	7463	t1	SF	FT4, TSH, TPO-Ab, Tg-Ab	–	FT4: was ↓ in ID group and ↑ in IO group (both compared to normal mothers). TSH: was ↔ in ID group and ↓ in IO group (both compared to normal mothers). TPO-Ab: was ↑ in ID group (compared to normal mothers) Tg-Ab: ↔
Iqbal et al. (2019) (40)	Austria	CS	80	t3	Hb, SF	TT3, TT4, TSH	–	No association between thyroid function and iron status was reported.
Mogahed et al. (2019) (41)	Egypt	CS	180	t1, t2	Hb, SF	TSH, FT4	Subclinical hypothyroidism (t1: 34.6%, t2: 36.8%) Overt hypothyroidism (t1: 9.6%, t2: 10.5%) Hypothyroxinemia (t1: 1.9%, t2: 1.9%)	FT4: t1 (n = 104): ▴ with Hb, ─ with SF. t2 (n = 76): ─ with Hb, ▴ with SF. TSH: t1 (n = 104): ─ t2 (n = 76): ▾ with Hb and SF.
Chen et al. (2020) (61)	Taiwan	PC	993	t3	Hb, Hct, RBCs count, MCV, MCH, MCHC	TT3, FT4, TT4, TSH	–	No association between thyroid function and iron status was reported.
Mahadik et al. (2020) (62)	India	PC	198	t3	Hb	FT3, FT4, TSH	Subclinical hypothyroidism (5.6%) Overt hypothyroidism (3.5%) Subclinical hyperthyroidism (1.5%)	* Anemia (Hb < 10 g/dl) was associated with ↑ prevalence of hypothyroidism.
Novakovic et al. (2020) (63)	Serbia	PC	46	t2	Hb, Hct, RBCs count, MCV, MCH, MCHC	FT4, TSH	Hypothyroidism (50%)	* Hb and RBCs count were ↓ in hypothyroid mothers.
Supadmi et al. (2020) (42)	Indonesia	CS	37	t1, t2, t3	SF	FT4, TSH	Hypothyroidism (45.9%)	There was no significant difference in the prevalence of hypothyroidism between the mothers with and without ID.
Zhang et al. (2020) (43)	China	CS	1592	t2	SF	FT4, TSH, TPO-Ab, Tg-Ab	Subclinical hypothyroidism (9.2%)	* In mothers with ID, FT4 ↓ and TSH ↑. * ID was not a risk factor for subclinical hypothyroidism or increased TPO-ab.
Bulunc et al. (2021) (44)	Turkey	CS	30	t1, t3	Hb	FT4, TSH	–	No association between thyroid function and iron status was reported.
Chowdhury et al. (2021) (64)	India	PC	100	t1, t2, t3	Hb	FT3, TT3, FT4, TT4, TSH, TPO-Ab	Subclinical hypothyroidism (17%) Overt hypothyroidism (4%) Subclinical hyperthyroidism (1%)	TSH: ▾ Hb
Syeda Farha et al. (2021) (45)	India	CS	110	t1	Hb	FT3, FT4, TSH	–	FT3: ▴ Hb FT4: ▴ Hb TSH: ▾ Hb
Hamed et al. (2021) (46)	Iraq	CS	74	t1	Hb, SF	TT3, TT4, TSH	–	TT3: ▴ with Hb and SF TT4: ▴ with Hb and SF TSH: ▾ Hb
Hassan et al. (2021) (47)	Pakistan	CS	180	t1	Hb, SF	FT3, FT4, TSH	Hypothyroidism (13%) Hyperthyroidism (10.5%)	No association between thyroid function and iron status was reported.
Hu et al. (2021) (70)	China	RC	14043	t1, t2	Hb, RBCs count	FT3, FT4, TSH	–	No association between thyroid function and iron status was reported.
Lisowska-Myjak et al. (2021) (65)	Poland	PC	65	t1, t2, t3	Tf	FT3, FT4, TSH	–	FT3: t1 (n = 55): ─ Tf t2 (n = 42): ─ Tf t3 (n = 39): ─ Tf FT4: t1 (n = 55): ▾ Tf t2 (n = 42): ─ Tf t3 (n = 39): ▾ Tf TSH: t1 (n = 55): ─ Tf t2 (n = 42): ─ Tf t3 (n = 39): ─ Tf
Moreno-Reyes et al. (2021) (48)	Belgium	CS	1241	t1, t3	Hb, SF, MCV, TfR, BIS	FT3, FT4, TT4, TSH, TPO-Ab, Tg-Ab, Tg	–	FT3: t1 (n = 594): ▴ with Hb, TfR t2 (n = 647): ▴ with Hb, SF * In mothers with anemia (Hb < 10 g/L), FT3 levels were ↓. FT4: t1 (n = 594): ▴ with Hb, SF, BIS t2 (n = 647): ▴ with Hb * In mothers with anemia (Hb < 10 g/L) or negative BIS, frequency of low FT4 was ↑ in t3. * In mothers with negative BIS, FT4 levels were ↓ in t3. TT4: t1 (n = 594): ▴ with SF, BIS t2 (n = 647): ▴ with Hb TSH: t1 (n = 594): ▾ with TfR t2 (n = 647): ─ Tg: t1 (n = 594): ─ t2 (n = 647): ─
Nie et al. (2021) (36)	China	CS	1761	t1	Hb	FT3, FT4, TSH	Subclinical hypothyroidism (3.7%) Subclinical hyperthyroidism (1.7%) Overt hyperthyroidism (0.01%) Overt hypothyroidism (0.01%)	FT3: ↔ FT4: ↔ TSH: was ↓ in mothers with mild anemia (10 < Hb <10.9 g/dl)
Zhu et al. (2021) (37)	China	RC	2378	t1	Hb, Hct	FT4, TSH, TPO-Ab	Subclinical hypothyroidism (9.1%)	* Anemia frequency was ↑ in mothers with subclinical hypothyroidism. * Anemia frequency was ↑ in mothers with TSH levels of > 4 mIU/l.
Wu et al. (2021) (50)	China	CS	4186	t2	Hb, SI	FT3, FT4, TSH	–	FT3: ▴ SI FT4: ▴ SI TSH: ▾ SI
Delcheva et al. (2022) (51)	Bulgaria	CS	61	t1, t2, t3	Hb, SF, TfR	FT4, TSH	–	FT4: ▴ with Hb and SF TSH: ─
Gupta et al. (2022) (12)	India	CS	100	t1	Hb, SF, SI, MCV	FT4, TSH, TPO-Ab	Subclinical hypothyroidism (26%)	FT4: ▴ SF TSH: ▾ SF TPO-Ab: ▾ SF * In mothers with ID, FT4 was ↓ and TSH, TPO-Ab, and subclinical hypothyroidism frequency were ↑.
Hamza et al. (2022) (52)	Iraq	CS	28	t3	Hb, RBCs count	TT3, TT4	–	No association between thyroid function and iron status was reported.
Sharifi et al. (2022) (53)	Iran	CS	130	t1	Hb, MCV, MCH, MCHC	TSH	–	TSH: ▾ with MCHC and ─ with Hb, MCV, MCH
Vinayagamoorthi et al. (2022) (6)	India	CS	144	t1	Hb, SF, SI	FT3, FT4, TSH, TPO-Ab, Tg-Ab	–	FT4: ▴ with Hb, SF, SI TSH: ▾ with Hb, SF, SI * In mothers with hypothyroidism, Hb, SF, and SI levels were ↓.
Wang et al. (2022) (54)	China	CS	2218	t1, t2, t3	Hb, SF	FT3, TT3, FT4, TT4, TSH	Hyperthyroidism (17.36%) Hypothyroidism (1.94%)	FT3: ▴ with Hb and SF TT3: ▴ with Hb FT4: ▴ with Hb and SF TT4: ▴ with Hb and SF TSH: ▾ with Hb and SF * In mothers with ID or IDA, levels of FT3 and FT4 were ↓, while level of TSH and frequency of hypothyroidism were ↑.
Jain et al. (2023) (17)	India	PC	100	t1, t2	Hb	FT3, FT4, TSH	–	TSH: ▾ Hb * In hypothyroid mothers, Hb levels were ↓.
Noshiro et al. (2023) (66)	Japan	PC	99	t2, t3	SF, SI, TIBC	TT4, TSH	–	No association between thyroid function and iron status was reported.
Savitha et al. (2023) (16)	India	CS	491	t1	Hb, SF	TSH, TT3, TT4, TPO-Ab	Subclinical hypothyroidism (29.9%) Overt hypothyroidism (1.8%) Hyperthyroxinemia (1.4%)	* The TT3, TT4, TSH, and TPO-Ab levels of ID mothers had no difference compare with non-ID mothers.

↑, significantly higher; ↔, no significant differences; ↓, significantly lower; ▴, positive correlation; ─, no significant correlation; ▾, negative correlation.

BIS, body iron store; CS, cross-sectional; DIR, dietary intake recall; EP, erythropoietin; FT3, free tri-iodothyronine; FT4, free thyroxine; Hb, hemoglobin; Hct, hematocrit; ID, iron deficiency; IDA, iron deficiency anemia; IO, iron overload; MCH, mean corpuscular hemoglobin; MCHC, mean corpuscular hemoglobin concentration; MCV, mean corpuscular volume; n, number; PC, prospective cohort; PCV, packed cell volume; RBC, red blood cell; RC, retrospective cohort; r-T3, reverse-tri-iodothyronine; SF, serum ferritin; SI, serum iron; T3, tri-iodothyronine; T4, thyroxine; TBG, thyroid-binding globulin; TBI, total body iron; Tf, transferrin; TfR, transferrin receptor; Tg, thyroglobulin; Tg Ab, anti-thyroglobulin antibody; TIBC, total iron-binding capacity; TPO-Ab, anti-thyroid peroxidase antibody; t, trimester; TS, transferrin saturation; TSH, thyroid stimulatory hormone; TT3, total tri-iodothyronine; TT4, total thyroxine; UIBC, urinary iron-binding capacity; UP, urinary perchlorate level. * represents additional information.

### 3.3 Quality assessment

The quality assessment results of the included studies are presented in **Table 2**. A total of 32 studies were deemed eligible for inclusion in further meta-analyses. Four studies were classified as having very good overall quality (29, 32, 48, 58), employing well-designed and mature study designs and reporting styles. Additionally, 23 studies demonstrated good overall quality (6, 12, 16, 28, 30, 33, 34, 36, 38–40, 43–45, 49, 52–54, 56, 59, 61, 66, 70), while five studies were rated as having satisfactory overall quality (41, 42, 46, 47, 51).

**Table 2:** Risk of bias assessment of the included studies based on the Newcastle-Ottawa Quality Assessment Scale (adapted for cross-sectional studies).

Studies	Selection				Comparability	Outcome		Quality score^*^
	Representativeness of the sample	Sample size	Non- respondents	Ascertainment of exposure	Based on design and analysis	Assessment of outcome	Statistical test	
Price et al. (2001) (56)	*		*	**	*	**	*	8
Zimmermann et al. (2007) (28)	*	*	*	**		**	*	8
Mahajan et al. (2008) (58)	*	*	*	**	*	**	*	9
Jaiswal et al. (2014) (29)	*	*	*	**	*	**	*	9
Joshi et al. (2014) (30)	*		*	**		**	*	7
Yu et al. (2015) (32)	*	*	*	**	*	**	*	9
DeZoysa et al., 2016) (59)	*		*	**		**	*	7
Li et al. (2016) (33)	*		*	**		**	*	7
Veltri et al. (2016) (34)	*	*		**	*	**	*	7
Fu et al. (2017) (36)	*		*	**		**	*	7
He et al. (2018) (38)	*		*	**	*	**	*	8
Zhang et al. (2019) (39)	*		*	**	*	**	*	8
Iqbal et al. (2019) (40)			*	**	*	**	*	7
Mogahed et al. (2019) (41)			*	**		**	*	6
Chen et al. (2020) (61)	*		*	**		**	*	7
Supadmi et al. (2020) (42)			*	**		**		5
Zhang et al. (2020) (43)	*		*	**	*	**	*	8
Bulunc et al. (2021) (44)		*	*	**		**	*	7
Syeda Farha et al. (2021) (45)	*		*	**		**	*	7
Hamed et al. (2021) (46)			*	**		**	*	6
Hassan et al. (2021) (47)	*		*	**		*		5
Hu et al. (2021) (70)	*		*	**	*	**	*	8
Moreno-Reyes et al. (2021) (48)	*	*	*	**	*	**	*	9
Nie et al. (2021) (49)	*			**	*	**	*	7
Delcheva et al. (2022) (51)			*	**		**	*	6
Gupta et al. (2022) (12)	*		*	**		**	*	7
Hamza et al. (2022) (52)			*	**	*	**	*	7
Sharifi et al. (2022) (53)			*	**	*	**	*	7
Vinayagamoorthi et al. (2022) (6)	*		*	**		**	*	7
Wang et al. (2022) (54)	*		*	**	*	**	*	8
Noshiro et al. (2023) (66)	*		*	**		**	*	7
Savitha et al. (2023) (16)	*		*	**		**	*	7

^*^ Studies with ≥7 points were considered high quality. The asterisks denote the score(s) for each criterion. One asterisk denotes a score of 1, and 2 asterisks denote a score of 2.

### 3.4 Meta-analysis

#### 3.4.1 Maternal SF concentration during pregnancy and its association with thyroid hormones

**Figure 2:**
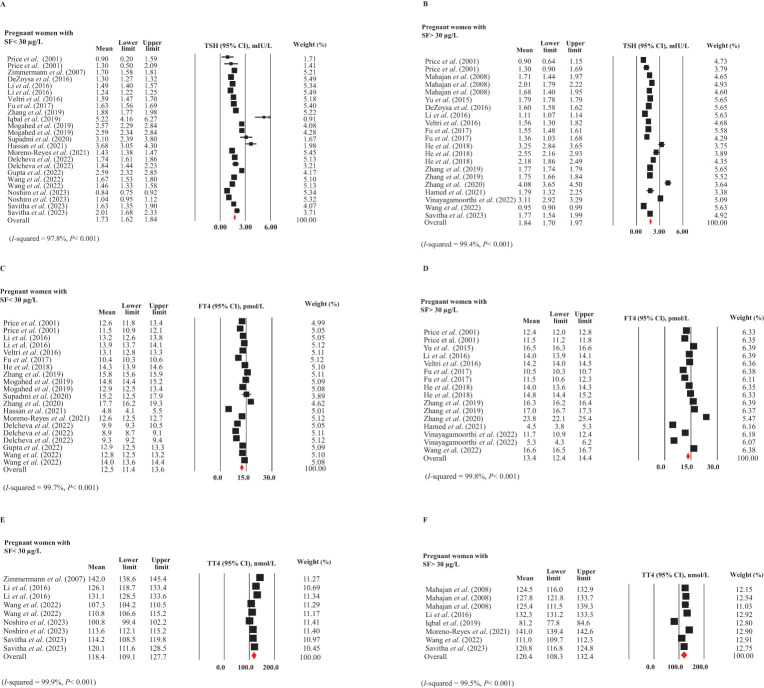
Forest plots for meta-analyses estimating the pooled thyroid hormones in pregnant women with (SF < 30 µg/L) and without (SF > 30 µg/L) ID. **(A)** TSH in ID, **(B)** TSH in non-ID, **(C)** FT4 in ID, **(D)** FT4 in non-ID, **(E)** TT4 in ID, and **(F)** TT4 in non-ID.

**Figures 2A, B** present the pooled mean and 95% CI of TSH levels in pregnant women with and without ID, based on SF concentrations. These plots indicated substantial heterogeneity between the study-specific estimates (test for heterogeneity: *I*² = 97.8% and 99.4%, respectively, P < 0.001 for both), necessitating the use of a random-effects model for a more appropriate estimate. No publication bias was detected between these two subgroups. The mean TSH levels in pregnant women with ID (SF < 30 μg/L) (12, 16, 28, 33, 34, 36, 39–42, 47, 48, 51, 54, 56, 59, 66) was 1.73 mIU/L (95% CI: 1.62-1.84 mIU/L), while in pregnant women without ID (SF > 30 μg/L) (6, 16, 32–34, 36, 38, 39, 43, 46, 54, 56, 58, 59) it was 1.84 mIU/L (95% CI: 1.70-1.97 mIU/L). Comparing the 95% CIs, no significant difference was found in TSH levels between pregnant women with and without ID.

In **Figures 2C, D**, the forest plots show the mean and 95% CIs for FT4 levels in pregnant women with ID (12, 33, 34, 36, 38, 39, 41–43, 47, 48, 51, 54, 56) and without ID (6, 32–34, 36, 38, 39, 43, 46, 54, 56), along with the pooled estimates. The studies included for women with and without ID exhibited substantial heterogeneity (*I*² = 99.7% and 99.8%, respectively, P < 0.001 for both). There was no publication bias in these subgroups. The mean FT4 levels in pregnant women with ID were 12.5 pmol/L (95% CI: 11.4-13.6 pmol/L), while in those without ID, the levels were 13.4 pmol/L (95% CI: 12.4-14.4 pmol/L). Comparing the 95% CIs indicated that these differences were not statistically significant.

The pooled TT4 (95% CI) of pregnant women with (16, 28, 33, 54, 66) and without ID (16, 33, 40, 48, 54, 58) are given in **Figures 2E, F**. The studies included for women with and without ID showed substantial heterogeneity (*I* ^2^ = 99.9 and 99.5%, respectively, *P <*0.001 for both). We observed no publication bias in these subgroups. There was no significant difference in TT4 levels between pregnant women with ID (118.4 nmol/L, 95% CI: 109.1-127.7 nmol/L) and those without ID (120.4 nmol/L, 95% CI: 108.3-132.4 nmol/L).

The funnel plots for publication bias assessment are provided in the **Supplementary Materials**.

#### 3.4.2 Maternal Hb concentration during pregnancy and its association with thyroid hormones

**Figure 3:**
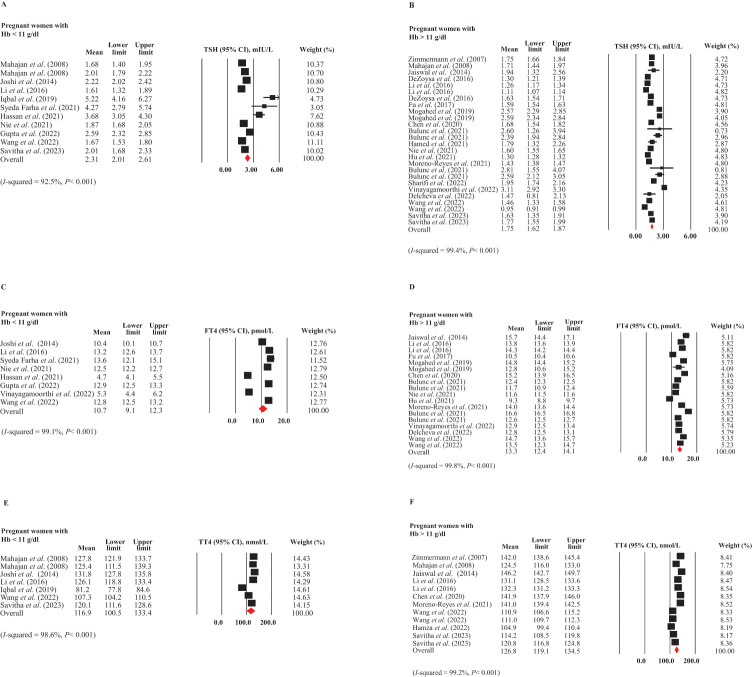
Forest plots for meta-analyses estimating the pooled thyroid hormones in pregnant women with (Hb < 11 g/dL) and without (Hb > 11 g/dL) ID. **(A)** TSH in ID, **(B)** TSH in non-ID, **(C)** FT4 in ID, **(D)** FT4 in non-ID, **(E)** TT4 in ID, and **(F)** TT4 in non-ID.

In **Figures 3A, B**, the forest plots depict studies with mean values and 95% CIs, as well as the pooled estimates for the mean TSH in pregnant women with (12, 16, 30, 33, 40, 45, 47, 49, 54, 58) and without ID (6, 16, 28, 29, 33, 36, 41, 44, 46, 48, 49, 51, 53, 54, 58, 59, 61, 70) based on Hb concentration. The plots showed substantial heterogeneity among included studies (*I* ^2^ = 92.5 and 99.4%, respectively, *P*<0.001 in both), necessitating the use of a random-effects model. The mean TSH levels were higher in pregnant women with ID (Hb < 11 g/dL) compared to those without ID (Hb > 11 g/dL). A comparison of the 95% CIs revealed a significant difference between these values [2.31 mIU/L (95% CI: 2.01-2.61 mIU/L) vs. 1.75 mIU/L (95% CI: 1.62-1.87 mIU/L)]. After applying the trim and fill method to correct for publication bias in the subgroup of pregnant women without ID (P = 0.004), the mean TSH level in women without ID was adjusted to 1.67 mIU/L (95% CI: 1.54-1.79 mIU/L). This difference between the two subgroups remained statistically significant even after bias correction.

Regarding FT4 levels, due to substantial heterogeneity among the included studies (I² = 92.5% and 99.4%, respectively, P < 0.001 for both), a random-effects model was employed for the analysis. As depicted in **Figures 3C, D**, the weighted FT4 levels (95% CI) were 10.7 pmol/L (95% CI: 9.1-12.3 pmol/L) in pregnant women with ID (6, 12, 30, 33, 45, 47, 49, 54) and 13.3 pmol/L (95% CI: 12.4-14.1 pmol/L) in those without ID (6, 29, 33, 36, 41, 44, 48, 49, 51, 54, 61, 70), indicating a significant difference between the two subgroups. There was no publication bias in these subgroups.

As illustrated in **Figures 3E, F**, substantial heterogeneity was observed between the two subgroups of studies (I² = 98.6% and 99.2%, respectively, P < 0.001 for both). The mean values and 95% CIs for TT4 levels were 116.9 nmol/L (95% CI: 100.5-133.4 nmol/L) in pregnant women with ID (16, 30, 33, 40, 54, 58) and 126.8 nmol/L (95% CI: 119.1-134.5 nmol/L) in those without ID (16, 28, 29, 33, 48, 52, 54, 58, 61). However, this difference was not statistically significant. No publication bias was detected in these two groups.

The funnel plots for publication bias assessment are provided in the **Supplementary Materials**.

### 3.5 Meta-regression

**Figure 4:**
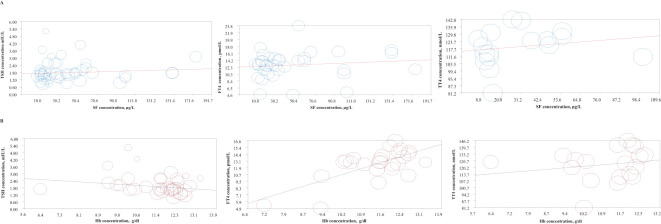
Meta-regression plots examining the association between thyroid hormone levels (TSH, FT4, and TT4) with **(A)** SF levels, and **(B)** Hb levels during pregnancy.

The meta-regression analyses indicated no significant associations between maternal SF and TSH (P = 0.081), FT4 (P = 0.246), or TT4 (P = 0.524) levels during pregnancy (**Figure 4A**). As shown in **Figure 4B**, there was a significant inverse association between maternal Hb and TSH levels (pooled β (SE) = -0.119 (0.046), P = 0.009). However, a significant positive association was observed between Hb and FT4 concentrations (pooled β (SE) = 1.395 (0.255), P < 0.001). No significant association was found between Hb and TT4 levels in pregnant women (P = 0.419). Detailed results of the meta-regression analyses are presented in **Table 3**.

**Table 3:** Results of the meta-regression analyses on the association of SF and Hb levels with thyroid hormone levels during pregnancy.

Iron status indicator	Thyroid hormone	Pooled β	Lower limit	Upper limit	Standard error	P-value
SF	TSH	0.002	-0.000	-0.004	0.001	0.081
	FT4	0.009	-0.007	0.027	0.008	0.246
	TT4	0.114	-0.238	0.467	0.179	0.524
Hb	TSH	-0.119	-0.211	-0.029	0.046	0.009*
	FT4	1.395	0.895	1.894	0.255	< 0.001*
	TT4	1.951	-2.782	6.684	2.415	0.419

FT4, free T4; Hb, hemoglobin; SF, serum ferritin; TSH, thyroid-stimulating hormone; TT4, total T4.

* Statistically significant association was observed (P-value < 0.05).

**Figure 5:**
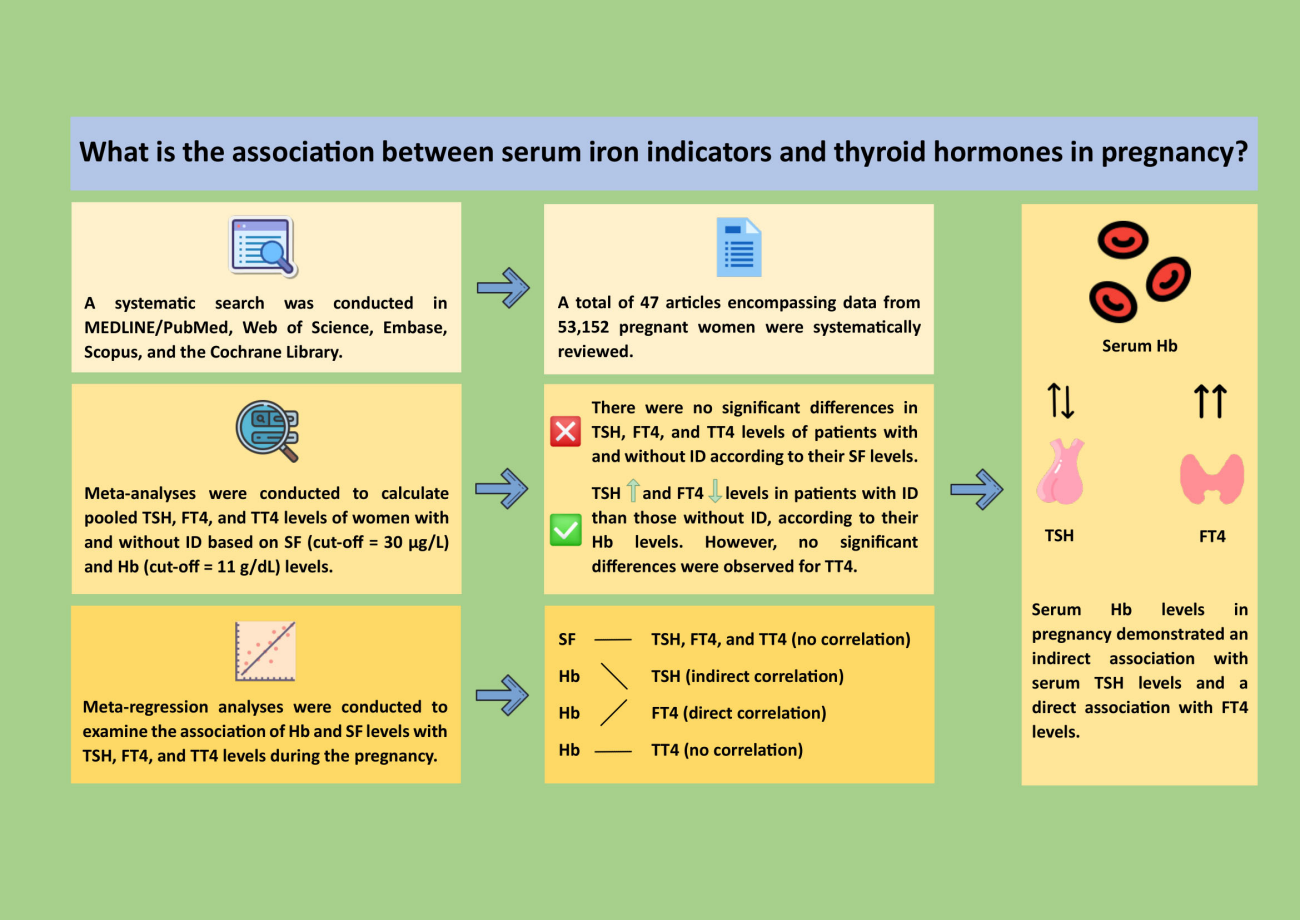
(Graphical abstract): A systematic review of studies from MEDLINE/PubMed, Web of Science, Embase, Scopus, and Cochrane Library up to 2023 was performed. Meta-analyses assessed pooled thyroid hormone levels with serum ferritin (SF, cut-off = 30 µg/L) and hemoglobin (Hb, cut-off = 11 g/dL). Forty-seven studies with 53,152 pregnant women were included. No significant differences in TSH, FT4, or TT4 were found for SF levels. However, iron deficiency was linked to higher TSH (2.31 vs. 1.75 mIU/L) and lower FT4 (10.7 vs. 13.3 pmol/L) but not TT4. Meta-regression showed maternal Hb levels had significant associations with TSH and FT4, but not TT4. These results suggested that monitoring maternal serum Hb may improve early detection and management of thyroid dysfunction during pregnancy (Icons by icons8.com). FT4, free T4; Hb, hemoglobin; ID, iron deficiency; SF, serum ferritin; TSH, thyroid-stimulating hormone; TT4, total T4.

Furthermore, a graphical abstract for our review findings is provided in **Figure 5**.

## 4 Discussion

Our meta-analyses revealed that defining ID based on Hb levels in pregnant women was associated with a higher likelihood of thyroid dysfunction, evidenced by elevated TSH levels and reduced FT4 concentrations, compared to those with sufficient iron status. However, no significant differences were observed in TT4 levels. In contrast, when maternal SF levels were used as the marker of ID, no significant differences in TSH, FT4, or TT4 levels were observed between pregnant women with and without ID. These findings were further corroborated by our meta-regression analyses, which demonstrated stronger associations between Hb levels and various thyroid hormones compared to SF.

Iron is a crucial trace element, essential for intracellular oxygen transport and the proper functioning of various enzymes (71). Given iron’s significant role in intracellular oxygen delivery and its involvement as a component of the TPO enzyme, which catalyzes iodine oxidation—the initial step in thyroid hormone production—it is plausible that reduced SI levels and related indicators may be significantly associated with thyroid dysfunction (12). In this regard, previous research highlighted the association between the iron profile and thyroid function. Previous research has underscored the relationship between iron status and thyroid hormone levels. A large cross-sectional study involving 42,162 participants demonstrated that individuals with hypothyroid or hyperthyroid function were more likely to have anemia. Furthermore, baseline thyroid dysfunction was associated with an increased likelihood of developing anemia during follow-up (72). Another study, conducted among 2,356 participants in the United States, revealed an inverse relationship between iron status and thyroid autoimmunity in reproductive-aged women (72). Specifically, each unit increase in SI was linked to a 43% reduction in the risk of Hashimoto’s thyroiditis (73). Additionally, SI was found to negatively correlate with TPO-Ab and exhibit a non-linear association with Tg-Ab level (73). Moreover, a recent meta-analysis of ten cross-sectional studies further highlighted this interplay, showing that patients with ID had significantly lower levels of TSH, FT4, and FT3 compared to those without ID (74). This suggests a potential connection between iron deficiency, thyroid function, and thyroid autoimmunity, particularly in certain patient groups. Notably, the meta-analysis emphasized a stronger association between these factors in pregnant women (74), underscoring the need for a deeper understanding of the interactions between iron metabolism and thyroid function in this population.

Our findings indicated a significant association between maternal iron status, specifically Hb levels, with thyroid function during pregnancy. Interestingly, we observed that serum Hb levels are a superior predictor of thyroid function during pregnancy compared to SF levels. Although previous research has acknowledged serum Hb as a widely available and cost-effective indicator of iron status, it has also highlighted its significant limitations, particularly its reduced sensitivity and specificity in detecting ID (75). On the other hand, SF has traditionally been regarded as a more specific and reliable indicator of iron status, as it provides insights into the size of iron stores in the body. However, the utility of SF as an iron status marker may be compromised during life stages such as pregnancy, where iron stores are physiologically depleted (75). Moreover, SF measures the amount of stored iron rather than the oxygen-carrying capacity of blood, which is directly reflected by Hb levels (76, 77). Given the critical role of iron in the oxygenation processes involved in thyroid hormone synthesis (78–80), it is rational to observe a stronger association between Hb levels and thyroid function compared to SF levels This suggests that during pregnancy, when iron demands are heightened, Hb may serve as a more direct and relevant marker for assessing thyroid function.

This review possesses several key strengths that distinguish it from other studies examining the association of serum Hb and SF levels with thyroid function during pregnancy (9, 74). First, we conducted an extensive and systematic search for eligible studies that provided data on at least one iron indicator and one thyroid function indicator, allowing us to offer a comprehensive overview of the associations and comparisons among these indicators. Furthermore, we performed multiple meta-analyses to precisely assess the relationships between thyroid function indicators (TSH, FT4, and TT4) and the two prominent iron status indicators, Hb and SF. Our comprehensive search strategy enabled us to incorporate data from a large cohort of pregnant women, which enhances the generalizability of our findings to broader populations. This also allowed us to compare the pooled values and 95% CIs of TSH, FT4, and TT4 levels between women with and without ID. Additionally, by pooling data from all eligible studies, we conducted an in-depth meta-regression analysis, providing robust insights into the relationship between different iron status indicators and thyroid function tests during pregnancy, as well as their predictive capacities.

However, several limitations of this review should be acknowledged. A substantial proportion of the included studies were conducted in Eastern and Southern Asia, which may constrain the geographical generalizability of our findings. Additionally, the variation in study designs and measurement methods among the included studies resulted in significant heterogeneity across all meta-analyses. Furthermore, the populations studied encompassed patients from different trimesters of pregnancy, introducing additional variability. Consequently, further research is needed to obtain more reliable and definitive results regarding the association between maternal iron status and thyroid function, as well as to clarify the specific relationships and diagnostic values of iron indicators for thyroid function during each trimester of pregnancy.

In conclusion, this study established a significant association between maternal iron status and thyroid function during pregnancy, with serum Hb levels demonstrating stronger associations with thyroid indicators—specifically, an inverse relationship with TSH and direct relationships with FT4—compared to SF. These findings suggest that monitoring maternal Hb levels, which is a readily available and cost-effective test, can provide valuable insights into the potential risk of thyroid dysfunction during pregnancy. Early detection of such risks enables timely diagnostic and therapeutic interventions, which are crucial for preventing the serious adverse effects of thyroid dysfunction on both mothers and fetuses.

## Data Availability

The original contributions presented in the study are included in the article/**Supplementary Material**. Further inquiries can be directed to the corresponding authors.
