# Supplementary material for: Association of iron status indicators with thyroid hormone concentrations during pregnancy: a systematic review and meta-analysis
Source: Front Endocrinol (Lausanne). 2025 Jan 28;16:1533169. doi: 10.3389/fendo.2025.1533169 (PMC11816114; doi:10.3389/fendo.2025.1533169)
Supplement: Supplementary file 1 [file DataSheet1.docx]

**Figure S1.** Funnel plots showing the estimated missing studies identified by the trim-and-fill analysis (black dots) for meta-analyses of pooled thyroid hormones in pregnant women with ID (SF < 30 µg/L) and without ID (SF > 30 µg/L). Panels: (A) TSH in ID, (B) TSH in non-ID, (C) FT4 in ID, and (D) FT4 in non-ID.

**A B**

 **C D**

**Figure S2.** Funnel plots showing the estimated missing studies identified by the trim-and-fill analysis (black dots) for meta-analyses of pooled thyroid hormones in pregnant women with ID (Hb < 11 g/dL) and without (Hb > 11 g/dL) ID. Panels: (A) TSH in ID, (B) TSH in non-ID, (C) FT4 in ID, and (D) FT4 in non-ID.

**A B**

**C D**
